# Molecular characterization of mesenchymal stem cells in human osteoarthritis cartilage reveals contribution to the OA phenotype

**DOI:** 10.1038/s41598-018-25395-8

**Published:** 2018-05-04

**Authors:** Chathuraka T. Jayasuriya, Nan Hu, Jing Li, Nicholas Lemme, Richard Terek, Michael G. Ehrlich, Qian Chen

**Affiliations:** 1 Bone and Joint Research Center, The First Affiliated Hospital, Frontier Institute of Science and Technology, Xi’an Jiaotong University, Xi’an, 710054 China; 2 Department of Orthopaedics, Brown University/Rhode Island Hospital, Providence, RI 02906 USA

## Abstract

Adult human articular cartilage harbors a population of CD166+ mesenchymal stem cell-like progenitors that become more numerous during osteoarthritis (OA). While their role is not well understood, here we report that they are indeed part of cellular clusters formed in OA cartilage, which is a pathological hallmark of this disease. We hypothesize that these cells, termed OA mesenchymal stem cells (OA-MSCs), contribute to OA pathogenesis. To test this hypothesis, we generated and characterized multiple clonally derived stable/immortalized human OA-MSC cell lines, which exhibited the following properties. Firstly, two mesenchymal stem cell populations exist in human OA cartilage. While both populations are multi-potent, one preferentially undergoes chondrogenesis while the other exhibits higher osteogenesis potential. Secondly, both OA-MSCs exhibit significantly higher expression of hypertrophic OA cartilage markers COL10A1 and RUNX2, compared to OA chondrocytes. Induction of chondrogenesis in OA-MSCs further stimulated COL10A1 expression and MMP-13 release, suggesting that they contribute to OA phenotypes. Finally, knocking down RUNX2 is insufficient to inhibit COL10A1 in OA-MSCs and also requires simultaneous knockdown of NOTCH1 thereby suggesting altered gene regulation in OA stem cells in comparison to chondrocytes. Overall, our findings suggest that OA-MSCs may drive pathogenesis of cartilage degeneration and should therefore be a novel cell target for OA therapy.

## Introduction

Osteoarthritis (OA) is a common chronic disease characterized by a series of degenerative changes including articular cartilage degradation, osteophyte formation and subchondral bone sclerosis^1–6^. Articular chondrocytes were thought to be the only cell type in joint cartilage, whose senescence or death in the avascular and hypoxic environment contributes to cartilage degeneration during aging^7–9^. In recent years, it has been reported that mature articular cartilage contains a small population of mesenchymal stem cell (MSC)-like progenitors that are capable of differentiating into mature chondrocytes^10,11^. Furthermore, these cells exist in greater numbers in OA cartilage than normal cartilage tissues^12,13^. However, it is not clear why increasing numbers of these cells correlate with cartilage degeneration during OA. We observed in human OA cartilage tissue that these progenitor cells constitute OA cellular clusters, which is a well-established hallmark of this degenerative joint disease.

Hence we hypothesize that such progenitor cells in OA cartilage, herein termed OA mesenchymal stem cells (OA-MSC), may contribute to disease progression. This is in contrast to the paradigm that chondrogenic progenitor cells may contribute to tissue repair in OA cartilage^14–16^. As the first step to test this hypothesis, we isolated OA-MCSs and characterized them at the cellular and molecular levels in this study.

Relatively little is known about OA cartilage stem cell properties despite its existence as first shown more than ten years ago^17–19^. This is mainly due to the challenge to obtain adequate quantities of pure cell populations for detailed analysis. Following isolation from articular cartilage, these cells often need to be expanded due to their scarcity. For example, there is a persistent lack of a molecular marker set to define and distinguish OA-MSCs from other stem cell populations, such as bone marrow derived mesenchymal stem cells (BM-MSCs). Hence, it is unclear whether OA-MSCs are remnant MSCs residing in articular cartilage or an altogether distinct population of cells^20^. It is also unclear whether OA-MSCs are a uniform population of cells, or a mixed population consisting of several subsets that coexist in OA cartilage tissue^21^. Most importantly, it is not clear whether OA-MSCs have any specific properties to either contribute to or inhibit OA pathogenesis and progression.

In order to overcome these obstacles, we generated multiple clonally derived human OA-MSC cell lines from knee articular cartilage of human OA patients through stem cell isolation by fibronectin adhesion^10^. By characterizing these OA-MSCs at molecular and cellular levels, we were able to identify, for the first time, the novel properties of OA-MSCs including multiple stem cell populations with different chondrogenic and osteogenic potentials, elevated hypertrophic OA phenotypes, altered gene regulation, and stimulation of MMP-13 secretion after induction of chondrogenic differentiation.

## Results

### Mesenchymal stem cells contribute to cell clusters in human OA cartilage

**Figure 1:**
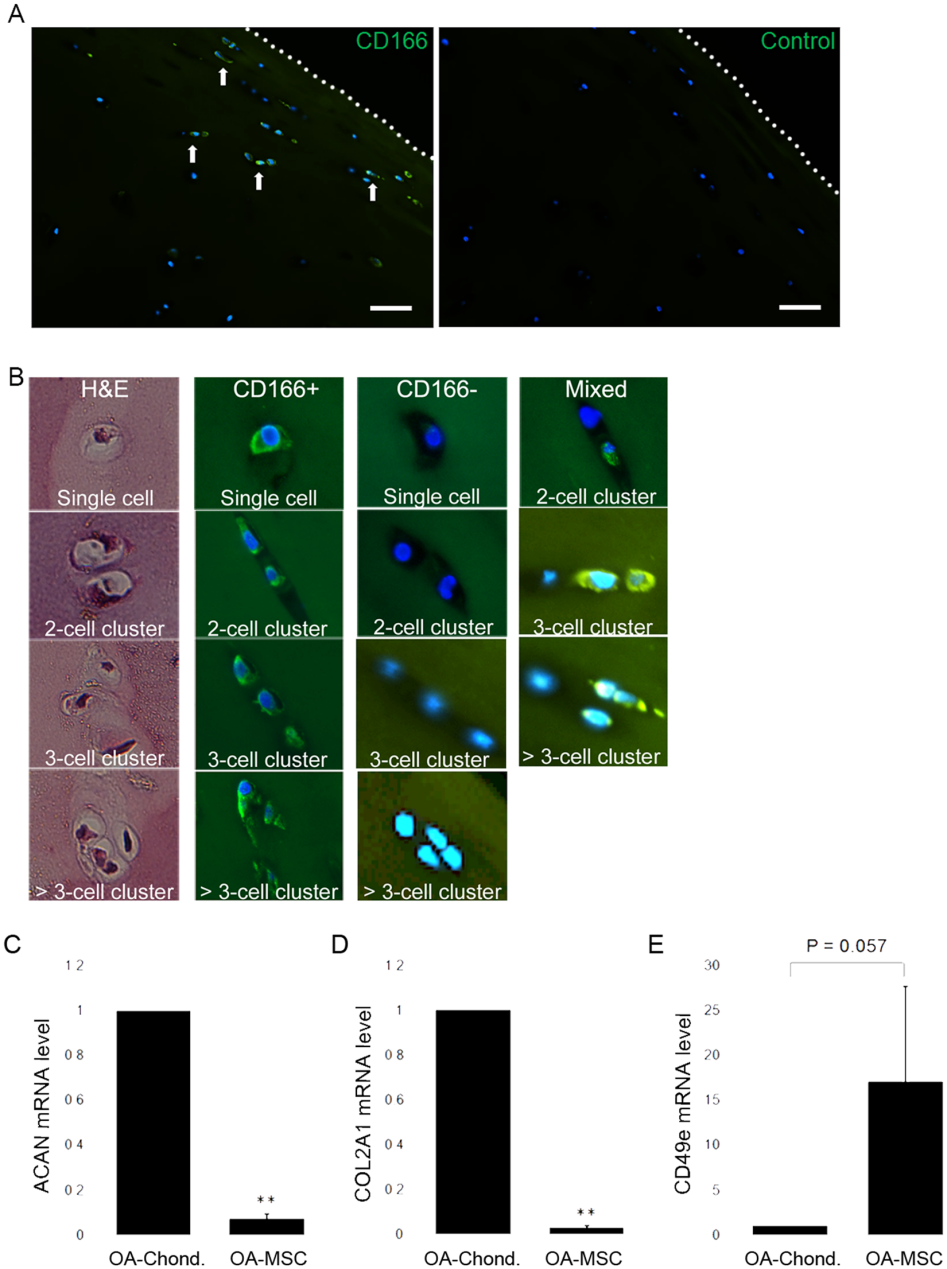
*In situ* analysis of primary OA cartilage derived mesenchymal stem cells and their gene expression. **(A)** Immunofluorescent staining of human OA cartilage sections stained with Dapi (blue) and an antibody against mesenchymal stem cell surface marker CD166 (green). White arrows indicate several positive staining events. The dotted line signifies the articular surface. Control section stained with Dapi and secondary antibody only. The dotted line signifies the articular surface. Scale bar represents 100 µm. **(B)** H&E staining and Immunoflorescent staining of human OA cartilage sections with Dapi (blue) and CD166 antibody (green) shows representative single OA stem cells as well as stem cell clusters (Left four panels). A cell cluster was defined as multiple cells occupying the same chondron. OA chondrocytes and chondrocyte clusters are CD166− (middle four panels). Clusters containing both OA chondrocytes and OA stem cells are labeled “mixed” and shown in the right most three panels. **(C)** Quantification of ACAN, **(D)** COL2A1 and **(E)** CD49e mRNA levels in CD166+ primary human OA cartilage stem cells, compared with CD166− primary human OA chondrocytes. n ≥ 3. **p ≤ 0.01, relative to CD166− chondrocytes. Quantitative data are represented as mean ± SD.

Cartilage samples of OA patients were sectioned and stained to visibly detect cells that express the membrane glycoprotein ALCAM (CD166), a progenitor/MSC marker that is not expressed by differentiated chondrocytes^22^ (Fig. 1A). Staining revealed that MSCs in OA cartilage largely reside in the superficial and intermediate tissue zones. These cells existed as either single cells, pure cell clusters (CD166+ cells only), or mixed clusters that also contain chondrocytes (Fig. 1B). A cell cluster is defined as multiple cells sharing the same pericellular matrix (i.e., chondron). The abundance of CD166+ cells and cell clusters ranged from 10.5% to 21.4% among total cell number in OA cartilage (Table 1). Since a hallmark of OA is the occurrence of cell clustering through clonal propagation in the superficial and intermediate zones of articular cartilage, we determined whether these CD166+ cells contribute to cell clustering in OA cartilage. We analyzed the abundance of CD166+ single cells as well as that of CD166+ cell clusters including 2-cell, 3-cell, and >3-cell clusters (Fig. 1B). The majority of these CD166+ cells existed in cell clusters, ranging from 51.3% to 76.2% of total CD166+ cells (Table 1). This percentage was comparable to that of CD166− cells (chondrocytes), whose percentage in CD166− clusters ranged from 54.5% to 75.4% among total CD166− cells in OA cartilage (Table 1). Thus, these CD166+ MSCs contribute to cell clusters in OA cartilage, which are herein referred as OA-MSCs.

**Table 1:** Percentage distribution of single and clustered cartilage stem cells (CD166+), single and clustered chondrocytes (CD166−), and combined clusters of stem cells and chondrocytes (CD166+/−) in osteoarthritis patient cartilage from femoral condyle.

	CD166+		CD166−		CD166+/−
	Single	Cluster	Single	Cluster	Cluster
Patient 1	2.5%	5.8%	22%	67.5%	2.2%
Patient 2	7.6%	7.6%	37.6%	46.8%	0.4%
Patient 3	7.2%	11.6%	35.8%	42.8%	2.6%

Clusters were defined as multiple cells occupying or appearing to occupy a single chondron. For each patient sample, cells in 4 independent fields of view (at 10X magnification) were individually counted to evaluate cell percentages.

By sorting for CD166+ cells using Fluorescently Activated Cell Sorting (FACS), OA-MSCs from human cartilage were isolated and enriched, and analyzed by real-time RT-PCR. OA-MSCs exhibited significantly lower expression of chondrogenic markers ACAN and COL2A1 mRNA and increased expression of integrin alpha 5 (ITGA5) fibronectin receptor (CD49e), in comparison to OA chondrocytes (Fig. 1C–E). Thus, OA-MSCs exhibit different molecular properties than OA chondrocytes. It also confirmed that fibronectin receptor is an appropriate selection marker for MSCs in OA cartilage.

### Multiple OA-MSC populations from human OA patients

**Figure 2:**
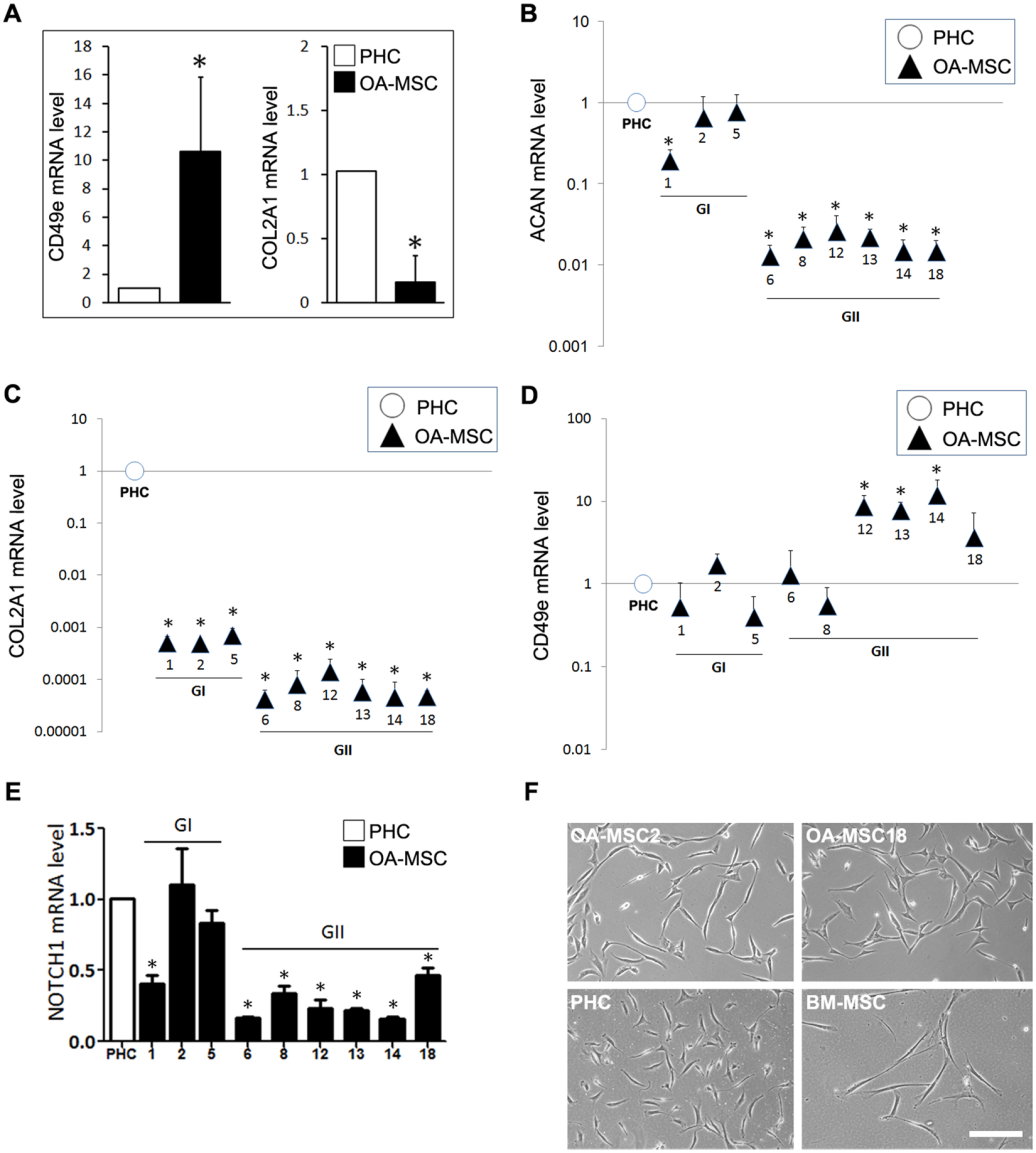
Gene expression and morphological analysis of human OA cartilage-derived stem cell lines. **(A)** Quantification of CD49e and COL2A1 mRNA levels in primary human osteoarthritis mesenchymal stem cells (OA-MSC), compared with primary human chondrocytes (PHC). **(B)** Relative aggrecan (ACAN), **(C)** type II collagen (COL2A1), **(D)** fibronectin receptor CD49e and **(E)** NOTCH1 mRNA expression levels in PHCs and nine OA-MSC lines. OA-MSC lines were categorized into two groups (GI, GII) based on their relative ACAN and COL2A1 expression levels. Due to the wide distribution of relative expression values, a logarithmic scale on the y-axis is implemented in B-D. **(F)** Cellular morphologies of OA-MSC2, OA-MSC18, PHCs and bone marrow derived mesenchymal stem cells (BM-MSCs). Images were acquired at an original magnification of 10× using an inverted microscope, Scale bar represents 100 µm. For all quantitative data, n ≥ 3. *p ≤ 0.05; **p ≤ 0.01, relative to PHCs. Quantitative data are represented as mean ± SD.

Taking advantage of their increased fibronectin receptor expression, compared to OA chondrocytes (Fig. 1E), primary OA-MSCs were enriched from the articular cartilage of human OA patients (age 50–75) through differential adhesion to fibronectin, as previously described^10^. The isolated OA-MSCs exhibited significantly higher mRNA levels (>16-fold) of ITGA5 the fibronectin receptor (CD49e) and lower levels (<0.05-fold) of chondrocyte marker type II collagen (COL2A1) than OA cartilage derived primary human chondrocytes (PHCs) (Fig. 2A). Individual OA-MSCs that had formed single colonies of ≥32 cells within one week were collected in the sequence from largest to smallest colonies (i.e. high to low colony-forming efficiency (CFE)). Colonies were grown and stabilized using retroviral gene transfer of Simian Virus 40 (SV40) encoded large T-antigen in order to efficiently produce cell numbers required to perform extensive molecular analysis. We sought to use these single cell-derived lines as a tool and resource for exploring the potential heterogeneity of OA-MSCs.

The resulting nine cell lines were further characterized by mRNA analysis of aggrecan (ACAN), COL2A1 and ITGA5 (CD49e). Similar to primary OA-MSCs, OA-MSC lines expressed lower COL2A1 and ACAN mRNA levels and higher CD49e levels than PHCs (Fig. 2B,C). Thus these OA-MSC lines retained the property of diminished chondrogenic marker expression that was exhibited by primary OA-MSCs. Furthermore, based on the expression levels of chondrogenic markers, they could be divided into two groups (Fig. 2B,C). Group I (GI) consisting of OA-MSC lines 1, 2 and 5, expressed higher levels of these chondrogenic markers, while Group II (GII) consisting of lines 6, 8, 12, 13, 14 and 18, expressed lower levels (Fig. 2B,C). GI OA-MSC lines expressed a similar level of ACAN as PHCs, while ACAN expression was reduced by up to 100-fold in GII OA-MSCs, in comparison with PHCs (Fig. 2B). COL2A1 expression was reduced by up to 2000-fold in GI OA-MSCs and up to 20,000-fold in GII OA-MSCs, in comparison to PHCs (Fig. 2C). Interestingly, GI contained the cell lines derived from individual OA-MSCs with higher CFE while GII contained the lines derived from those of lower CFE. Most GII cell lines expressed significantly higher CD49e mRNA levels (up to 10-fold), relative to the PHC control and the GI OA-MSC lines (Fig. 2D). This was consistent with primary OA-MSCs expressing higher CD49e mRNA levels than PHCs (Fig. 2A). It suggested that the OA-MSCs of lower CFE in GII were the main contributors of high CD49e mRNA levels in primary OA-MSCs. NOTCH1 mRNA expression levels in GI OA-MSC lines were generally comparable to that of PHCs with the exception of OA-MSC1 (Fig. 2E). OA-MSC lines in GII exhibited significantly lower expression of NOTCH1 than OA chondrocytes (Fig. 2E).

We compared the morphology in cell culture of OA-MSCs with primary human bone marrow derived MSCs (BM-MSCs), which were purchased from ATCC®. Despite differences in mRNA expression patterns between OA-MSCs in GI and GII, their cellular morphologies were similar as exemplified by the morphologies of OA-MSC2 (GI) and OA-MSC18 (GII) (Fig. 2F). BM-MSCs exhibited a long bipolar fibroblastic morphology whilst morphologies of OA-MSCs were that of an intermediate state (between BM-MSCs and chondrocytes) appearing to be more stellate in appearance (Fig. 2F).

To eliminate the possibility that differences between OA-MSCs in GI and GII (Fig. 2B–E) were simply due to individual patient variation, the patient origin of each cell line was determined by genetic profiling (Supplementary Table 1). The short tandem repeat (STR) profiles indicated that lines 1, 2, 5, 6, 12 and 18 originated from the same patient (female), whereas lines 8, 13 and 14 originated from another patient (male). Since OA-MSCs from different patients were distributed across each group (e.g., GII contained lines 6, 12, 18 from one patient and lines 8, 13, 14 from another patient), this verified that the observed differences in OA-MSCs between GI and GII were not attributed to individual patient variation.

### OA-MSCs are susceptible to hypertrophy, MMP-13 release and osteogenesis

**Figure 3:**
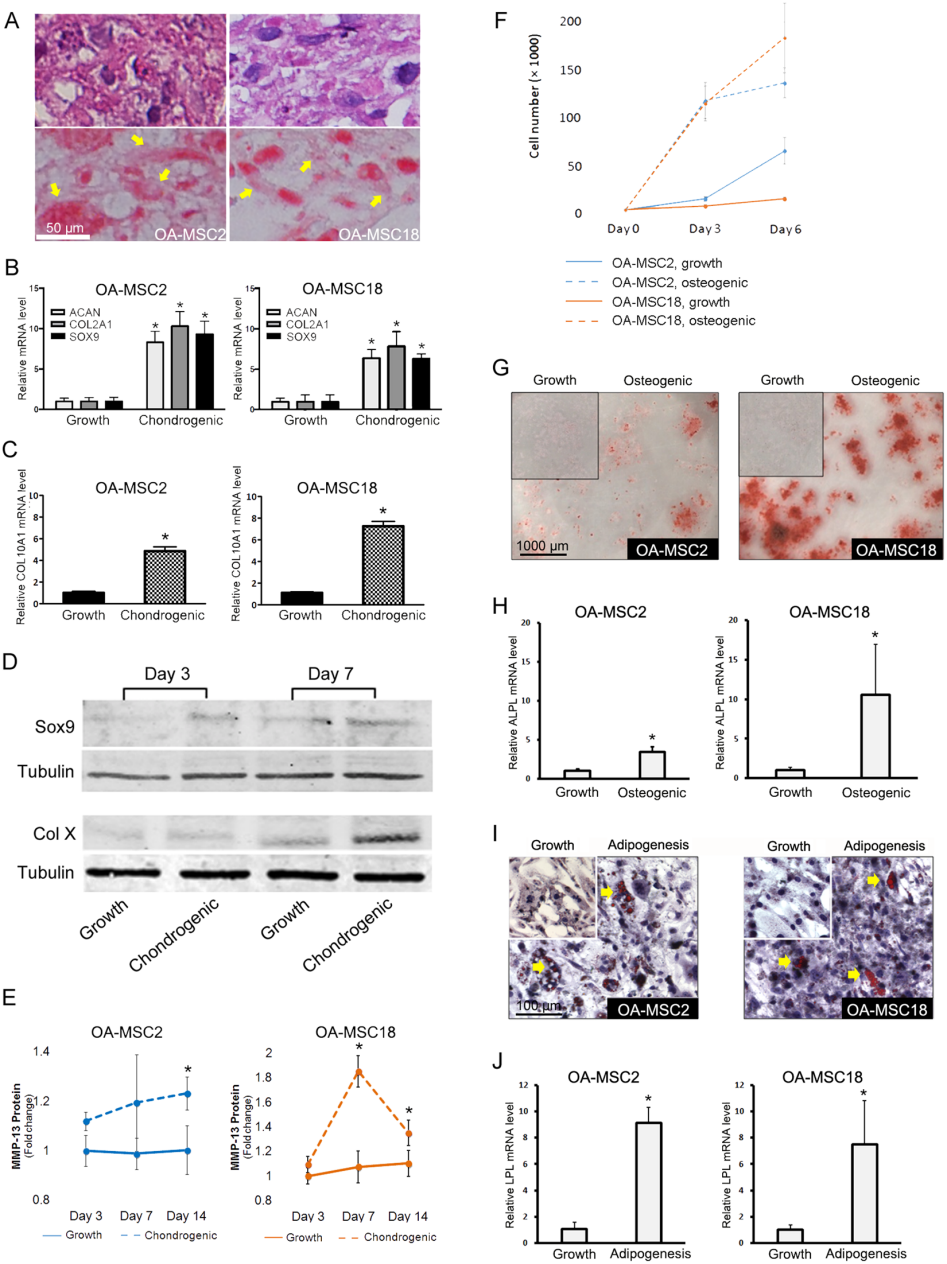
Differentiation analysis of clonal OA cartilage-derived MSCs. **(A)** Pellet cultures of OA-MSC2 and OA-MSC18 were sectioned, H&E stained (top panel) and Safranin O stained (bottom panel) after 21 days of induction with chondrogenesis medium and imaged at 20x magnification. Yellow arrows indicate areas of extracellular and pericellular matrix that stained positively for Safranin O. **(B)** ACAN, COL2A1, SOX9 mRNA expression levels and **(C)** COL10A1 mRNA expression level was quantified in monolayer culture following chondrogenesis induction. **(D)** Western blot analysis of SOX-9 and COL10 in OA-MSC2 after 3 days and 7 days in growth and chondrogenesis medium. Tubulin is used as a loading control. SOX-9 and respective tubulin loading control bands are from different parts of the same blot. COL10 and its respective tubulin loading control are from two different blots, but the loaded protein quantity was the same. **(E)** Active MMP-13 protein levels were quantified after 21 days in growth and chondrogenesis media by ELISA. **(F)** Cell proliferation rate of OA-MSC2 and OA-MSC18 was determined by quantifying viable cell number that results from culturing each cell line for 6 days in chondroprogenitor growth medium or osteogenesis induction medium. **(G)** OA-MSC2 and OA-MSC18 were stained with Alizarin Red following osteogenic induction for 21 days. **(H)** ALPL mRNA expression was quantified following osteogenesis induction. **(I)** Adipogenic induction of OA-MSC2 and OA-MSC18 for 21 days followed by Oil Red O staining. Yellow arrows indicate positive staining events. **(J)** LPL mRNA expression was quantified following adipogenesis induction. n ≥ 3. *p ≤ 0.05, relative to respective control groups cultured in growth media. Quantitative data are represented as mean ± SD.

To identify distinguishing features among different human OA-MSC populations, we randomly selected OA-MSC2 from GI and OA-MSC18 from GII for further characterization. We induced their differentiation under different culture conditions. Upon chondrogenesis induction in pellet culture, both OA-MSC2 and OA-MSC18 cells produced extracellular matrix (ECM) as indicated by H&E staining (Fig. 3A, top panel). Cells underwent chondrogenesis in pellet culture as indicated by positive Safranin-O staining (Fig. 3A, bottom panel). OA-MSC2 exhibited noticeably higher Safranin-O staining in the pellet culture. Upon induction of chondrogenesis by chondrogenesis medium in monolayer culture, chondrogenic markers ACAN, COL2A1 and SOX9 were elevated in both lines (Fig. 3B). Hypertrophic marker COL10A1 was elevated by approximately 5 and 7 fold in OA-MSC2 and OA-MSC18 respectively, indicating that these cells express markers of terminal differentiation found in hypertrophic cells under chondrogenic conditions (Fig. 3C). Western blot analysis indicated that SOX9 protein level was stimulated 3 days following chondrogenesis induction while Col X protein level was stimulated 7 days following chondrogenesis induction in OA-MSC2 (Fig. 3D). Chondrogenesis induction significantly increased the release of active MMP-13 from both OA-MSC2 and OA-MSC18, as quantified by ELISA (Fig. 3E).

In growth medium, OA-MSC2 had a three-fold higher cell proliferation rate (CPR) than OA-MSC18 (Fig. 3F), which was consistent with the higher CFE that was observed in the primary cartilage stem cells from which OA-MSC2 was generated. However, OA-MSC18 exhibited a more robust response to osteogenic induction than OA-MSC2. Induction of osteogenesis increased the CPR by 11.3 fold in OA-MSC18 by day 6 in comparison to the 2.1-fold CPR increase in OA-MSC2 (Fig. 3F). OA-MSC18 had stronger Alizarin Red staining (Fig. 3G) and a greater increase of alkaline phosphatase (ALPL) mRNA levels (11-fold) relative to the 4-fold increase in OA-MSC2 (Fig. 3H). Both cell lines exhibited moderate Oil Red O staining in response to adipogenic induction (Fig. 3I), and adipogenesis marker lipoprotein lipase (LPL) was elevated at similar levels (Fig. 3J). Thus, both lines were capable of a modest degree of adipogenesis upon induction.

### Cell surface markers that distinguish human OA-MSC populations

**Figure 4:**
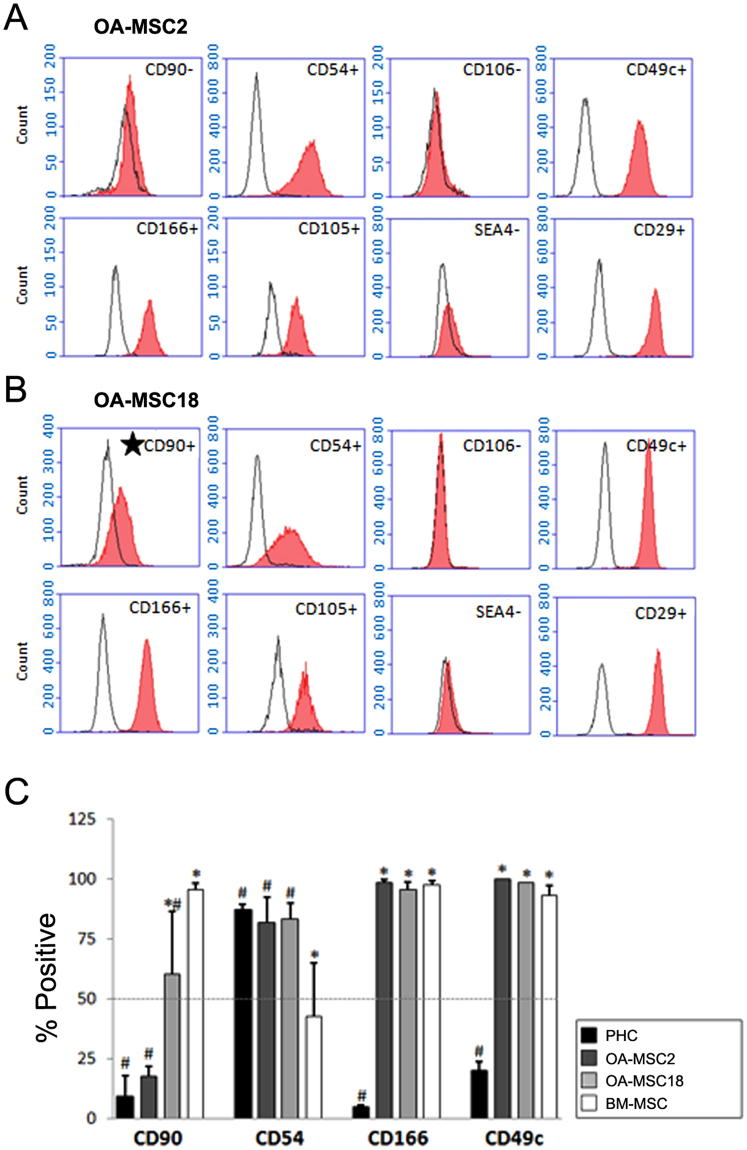
Cell surface marker analysis of clonal OA cartilage-derived MSCs. **(A)** Representative flow cytometry analysis of OA-MSC2 and **(B)** OA-MSC18. Empty peaks indicate the results obtained from cells stained with isotype control antibodies and filled peaks indicate the results of cells stained with the indicated specific target antibodies. **(C)** Histogram of compiled results of flow cytometry experiments using bone marrow derived mesenchymal stem cells (BM-MSCs), articular chondrocytes (PHCs), OA-MSC2 and OA-MSC18. n ≥ 3. ^#^p ≤ 0.05, relative to BM-MSCs. *p ≤ 0.05, relative to PHCs. Quantitative data are represented as mean ± SD.

OA-MSC2 and OA-MSC18 share common cell surface markers: both were positive for multiple MSC markers including CD29, CD49c, CD105, CD166, but negative for expression of SSEA4 – an early embryonic glycolipid antigen^23,24^ (Fig. 4A,B). Furthermore, they were both positive for CD54, a marker constitutively expressed by articular chondrocytes but lowly expressed by MSCs^25,26^, and negative for articular chondrocyte marker CD106^26^. Thus, common markers of OA-MSC are different from those of BM-MSC and articular chondrocytes, respectively.

The only surface marker that distinguished OA-MSC2 from OA-MSC18 was Thy-1 membrane glycoprotein (CD90). CD90 level was below 20% in OA-MSC2 (Fig. 4A,C), while above 60% in OA-MSC18 (Fig. 4B,C). Furthermore, there was a quantitative decrease of CD90 expression levels from BM-MSCs (97%), OA-MSC18 (62%), OA-MSC2 (18%), to PHCs (<10%) (Fig. 4C). Similar to BM-MSCs, both OA-MSCs shared high expression levels of CD49c and CD166 (Fig. 4C). On the other hand, similar to PHCs, both lines exhibited high expression levels of CD54 (Fig. 4C). Early passages (P1-P5) and late passages (P10-P20) of these lines exhibited consistent cell surface marker expression patterns (data not shown). Overall, these results indicate that OA-MSCs express a unique combination of cell surface markers from BM-MSC and articular chondrocytes that distinguished them from either cell type.

### OA-MSCs express high levels of hypertrophic markers in OA cartilage

**Figure 5:**
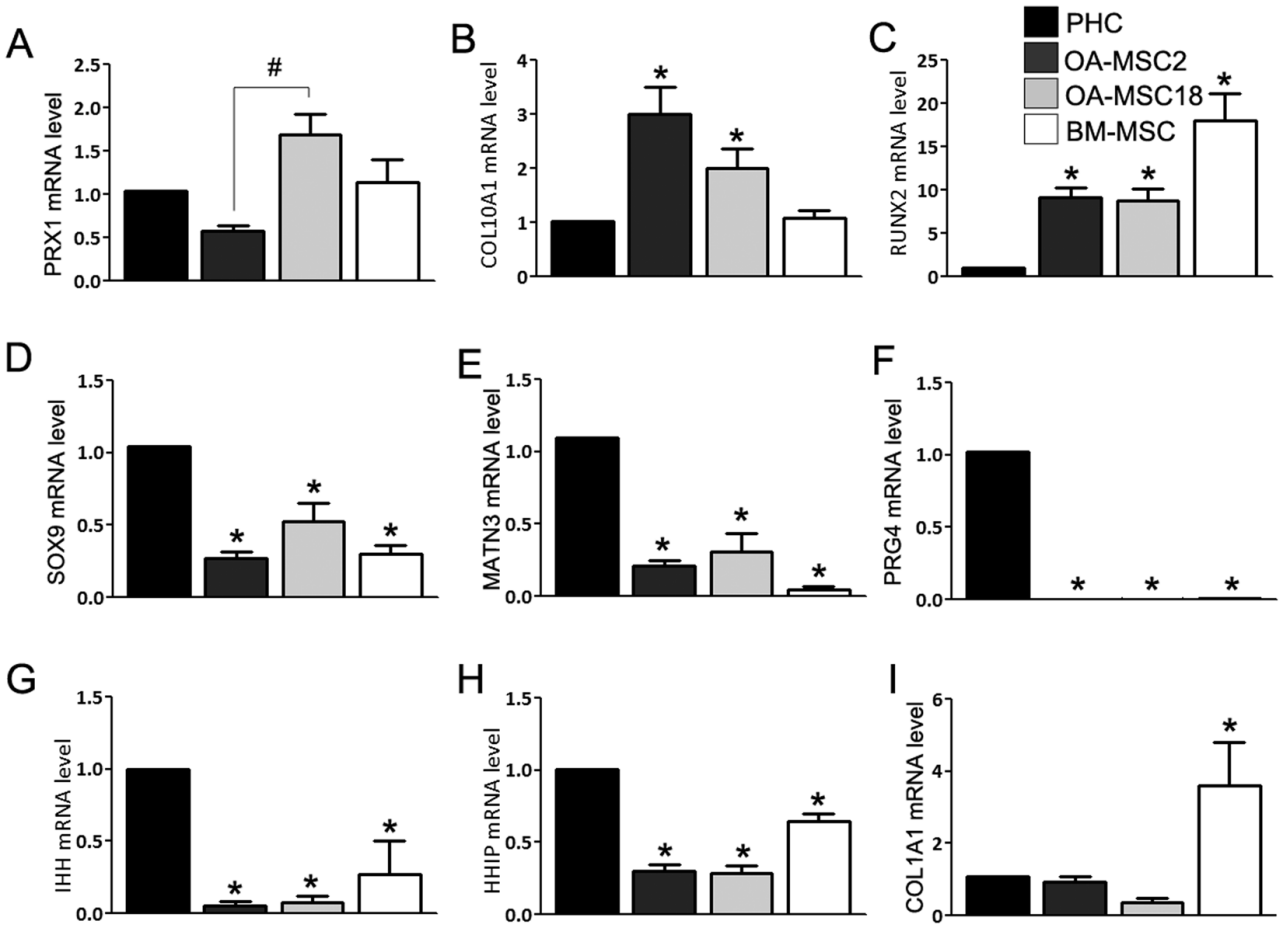
Comparison of gene expression profiles of clonal OA cartilage-derived MSCs, PHCs and BM-MSCs. **(A)** Relative mRNA expression mesenchymal transcription factor PRX1, **(B)** type X collagen (COL10A1), **(C)** RUNX2, **(D)** chondrogenesis transcription factor SOX9, **(E)** cartilage matrix protein matrilin-3, **(F)** cartilage surface lubricating protein PRG4, **(G)** Indian hedgehog (IHH), **(H)** hedgehog interacting protein (HHIP), **(I)** and stromal marker COL1A1, in primary human chondrocytes (PHCs), bone marrow mesenchymal stem cells (BM-MSCs), OA-MSC2 and OA-MSC18. n ≥ 3. *p ≤ 0.05, relative to the chondrocyte group. ^#^p ≤ 0.05. Data are represented as mean ± SD.

The other marker that distinguished different populations of OA-MSCs was PRX1, a limb bud mesenchymal cell transcription factor. PRX1 is a marker of cells in the mesenchymal lineage^27,28^. OA-MSC18 exhibited a 3-fold increase in PRX1 mRNA levels relative to OA-MSC2 (Fig. 5A).

Both OA-MSCs exhibited consistent elevation of terminal chondrocyte differentiation markers type X collagen (COL10A1) and RUNX2 mRNA, which are expressed by chondrocytes in OA cartilage (Fig. 5B,C). Strikingly, the expression levels of these markers were even higher than that of PHC from OA cartilage (Fig. 5B,C), which has been shown before to express higher levels of hypertrophic markers than normal chondrocytes^29–32^. Thus, OA-MSCs exhibit gene expression that is commonly associated with epiphyseal and hypertrophic chondrocytes.

Both OA-MSCs expressed significantly lower levels of chondrogenic markers than OA chondrocytes (Fig. 5D,E and F). These included chondrogenesis transcription factor SOX9, cartilage matrix protein MATN3, and cartilage lubrication protein lubricin (PRG4), a previously proposed marker of chondroprogenitor cells from articular cartilage^33^. OA-MSCs expressed significantly lower mRNA levels of Indian Hedgehog (IHH) and its downstream target hedgehog-interacting protein (HHIP) than PHCs from OA cartilage (Fig. 5G,H). On the other hand, similar to PHCs, both OA-MSCs expressed significantly lower levels of the stromal marker COL1A1 than BM-MSCs (Fig. 5I). Thus, COL1A1 can be used to distinguish both types of OA-MSCs from BM-MSCs.

### Altered gene regulation in OA-MSCs

**Figure 6:**
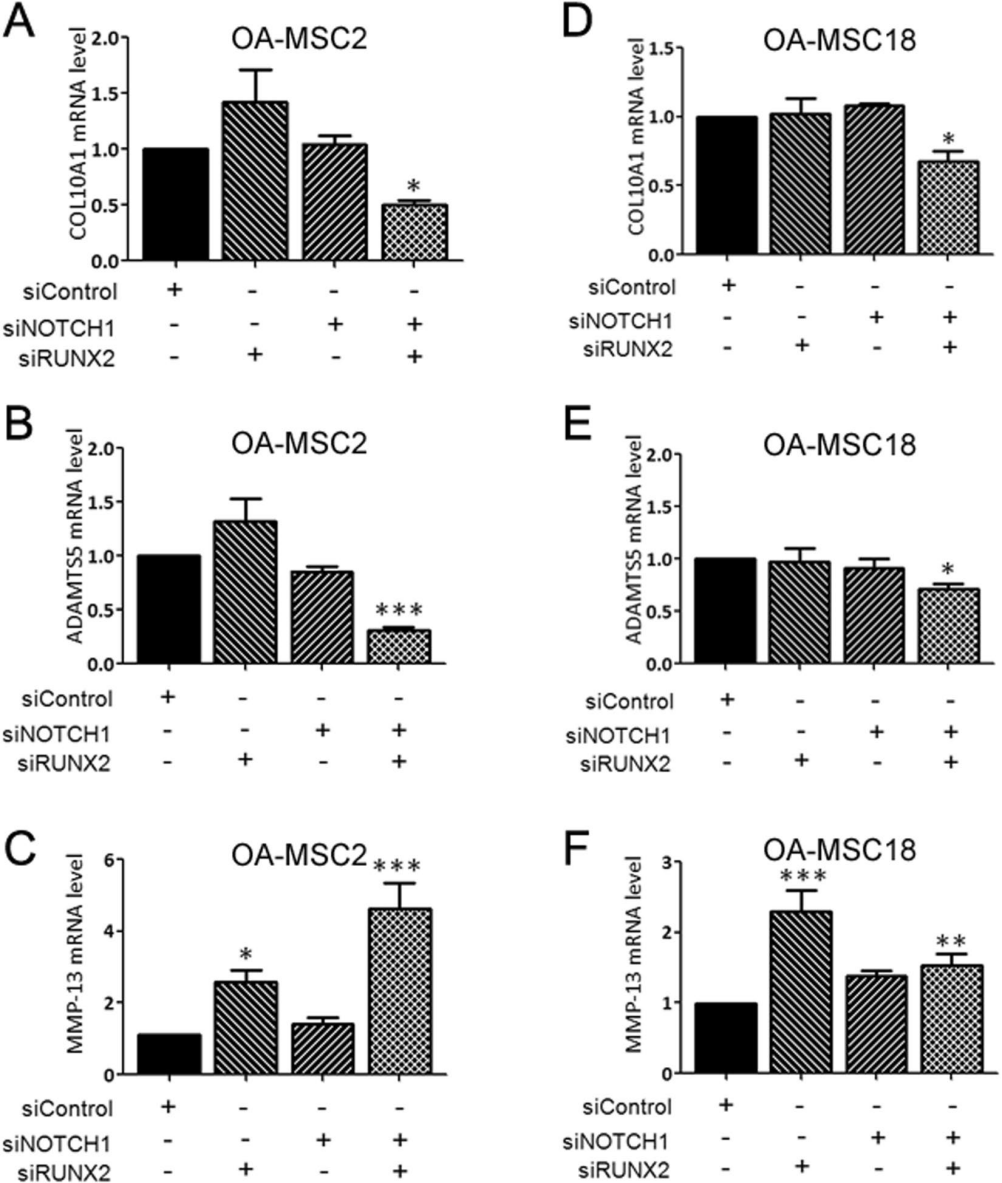
Knockdown of RUNX2 and NOTCH1 are required to attenuate type X collagen expression in clonal OA cartilage-derived MSCs. **(A–C)** mRNA expression of COL10A1, ADAMTS5 and MMP-13 following transient siRNA-based knockdown of RUNX2 and/or NOTCH1 in OA-MSCL2 and **(D–F)** in OA-MSCL18 using. n ≥ 3. **p ≤ 0.05, **p ≤ 0.01, ***p ≤ 0.005 relative to scrambled siRNA control transfected group. Data are represented as mean ± SD.

RUNX2 is a positive regulator of the chondrocyte epiphyseal phenotype during OA. It is a transcription factor that has been shown to be indispensable for chondrocyte hypertrophy^34^. It has been shown to promote hypertrophic markers including COL10A1 and MMP-13 in chondrocytes^35,36^. We next sought to suppress hypertrophic and other OA markers expression by targeted knockdown of RUNX2 in OA-MSCs (Supplementary Fig. 1). However, knockdown of RUNX2 alone failed to inhibit COL10A1 or ADAMTS5 expression in OA-MSC2 or OA-MSC18 (Fig. 6A,B,D,E). Furthermore, knocking down RUNX2 increased the expression of MMP-13 mRNA in OA-MSCs (Fig. 6C,F), which was the opposite of its effect in chondrocytes. Therefore, gene regulation pathways were altered in OA-MSCs in comparison to chondrocytes.

Since NOTCH1 has been shown to be involved in regulating chondrogenic progenitors and chondrocyte hypertrophy^37,38^, we next inhibited NOTCH1 expression by transfecting NOTCH1 siRNA in OA-MSCs (Supplementary Fig. 1). Knocking down NOTCH1 alone did not affect the expression of COL10A1, ADAMTS5, or MMP-13 in OA-MSCs (Fig. 6A–F). However, simultaneously knocking down RUNX2 and NOTCH1 significantly inhibited the expression of COL10A1 and ADAMTS5 (Fig. 6A,B,D,E). However, double knock-down of RUNX2 and NOTCH1 still resulted in significant increases of MMP-13 mRNA levels in OA-MSCs, similar to RUNX2 single knock-down (Fig. 6C,F).

## Discussion

OA cartilage harbors higher quantities of mesenchymal stem cell-like progenitor cells relative to normal healthy cartilage^12,13^. However, it is unknown whether these stem cells inhibit or contribute to OA pathogenesis, or remain inert in OA cartilage. The molecular and functional properties of these cells are unclear, because it is challenging to characterize them due to their low abundance and heterogeneity^12,13,18,19^. We overcame this challenge by generating and characterizing multiple cell lines each originating from different individual cartilage-derived stem cells isolated from OA patient cartilage. Cell lines were generated to efficiently produce a large population of human OA cartilage stem cells required to perform extensive cellular and molecular analysis. We further analyzed the properties of these cells in human OA cartilage. Our data revealed multiple novel properties of these stem cells that may contribute to several hallmark features of OA.

### Difference between OA-MSCs and OA chondrocytes

We find several distinctions between OA-MSCs from OA chondrocytes, which account for the majority of cells in OA cartilage. Firstly, their gene expression profiles are distinct from chondrocytes. Reduced COL2A1 and ACAN expression and increased CD49e^10^ expression are characteristic of all generated OA-MSC lines, compared to OA chondrocytes. Intriguingly, some cell lines exhibited higher expression of chondrocyte markers than did others. This result supports the notion that cell lines expressing higher chondrocyte markers originated from OA-MSCs that further differentiated along the chondrogenic lineage and are therefore characteristically more similar to chondrocytes than lines exhibiting lower expression of chondrocyte markers. Secondly, these OA-MSC lines do not express chondrocyte marker CD106^26^. Thirdly, matrilin-3 and SOX9 mRNA levels in OA-MSC lines were low, similar to that of BM-MSCs rather than OA chondrocytes. Both OA-MSC2 and OA-MSC18 exhibited low PRG4 expression, which has been proposed to be highly expressed by chondroprogenitor cells in the superficial zone during mouse articular cartilage development^33^.

### Difference between OA-MSCs and BM-MSCs

We also found characteristic features that distinguish these OA-MSCs from BM-MSCs. Although OA-MSCs share several common markers with BM-MSCs including CD29, CD49c, CD105 and CD166, they do not express SSEA4. In addition, OA-MSCs express high levels of CD54, which was lowly expressed by BM-MSCs but constitutively expressed by articular chondrocytes^25,26^.

### Multiple types of OA-MSCs

The present study reveals heterogeneity in OA cartilage-derived stem cells using clonal stem cell lines that originated from separate individual cells. It revealed at least two types of molecularly and functionally distinct OA-MSCs derived from adult human OA cartilage. We identified molecular and functional features of OA-MSCs that can be used to categorize them into different subsets. OA-MSC2 exhibited higher expression of chondrocyte markers (i.e. COL2A1 and ACAN) than OA-MSC18. However, OA-MSC18 expressed higher PRX1 mRNA levels (3-fold increase) over OA-MSC2. PRX1 expression has been reported to be elevated in cells that constitute the limb mesenchyme during skeletogenesis^39,40^. These mesenchymal precursors are particularly regarded for their plasticity during development. Hence, the higher PRX1 expression level in OA-MSC18 is consistent with our finding that this line also has higher plasticity than OA-MSC2. Furthermore, both of these cell lines had remarkably similar cell surface marker profiles including CD29+/CD49c+/CD54+/CD105+/CD106−/CD166+/SSEA4−. However, CD90 was expressed by a significantly higher proportion of cells in OA-MSC18, relative to OA-MSC2.

### OA-MSCs contain OA hallmarks

We present evidence implicating that OA-MSCs may fundamentally contribute to common pathogenic features of OA such as chondrocyte hypertrophy, osteogenesis, and release of degenerative matrix proteinase. Both types of OA stem cells express significantly higher levels of COL10A1 and RUNX2 mRNA, hypertrophic markers of OA cartilage, than OA chondrocytes. These data suggest that the OA-MSCs may be a strong contributor to elevating hypertrophic marker expression in OA cartilage.

Although both cell lines are multi-potent, OA-MSC18 undergoes more robust osteogenic differentiation upon induction. OA-MSC18 cells grew very slowly in growth medium, having less than one quarter of the proliferation rate of OA-MSC2. However, upon induction of osteogenesis, the proliferation rate of OA-MSC18 cells increased by 11.3-fold in comparison to the 2.1-fold increase in OA-MSC2. Thus, osteogenic conditions preferentially activate the growth of OA-MSC18 cells. During OA pathogenesis, the upregulation of inflammatory cytokines and production of bone morphogenic protein 2 (BMP2) by hypertrophic chondrocytes may promote an osteogenic microenvironment^41,42^. Our finding suggests that such an osteogenic microenvironment may activate some OA-MSCs to multiply and undergo osteogenesis. It is possible that the growth and osteogenesis of the OA-MSCs may contribute to the development of osteophytes, a hallmark of OA.

Another significant finding is that induction of chondrogenesis further stimulates COL10A1 expression and MMP-13 release in OA stem cells. It suggests that OA-MSCs contribute to OA phenotypes during cartilage repair. This is the opposite of the paradigm to activate stem cells for cartilage repair^14–16^. It indicates that once the articular cartilage reaches the OA stage, suppressing or deleting the OA-MSCs may be a better strategy to preserve the cartilage rather than activating OA-MSCs for repair. This is consistent with the recent demonstration to counter the degeneration process by deleting senescent cells from aging tissues^43^.

### Target OA-MSCs for OA therapy?

Cell clusters in articular cartilage have been recognized as a hallmark of OA and it is thought to be due to chondrocyte proliferation at the early stage of the disease^32,44^. We demonstrate here that OA-MSCs also contribute to cell clustering in OA cartilage. Some of these clusters consist entirely of CD166+ OA-MSCs. This suggests that OA stem cells undergo self-renewal and increase their percentage in OA cartilage. This may explain why the percentage of the CD166+ chondroprogenitor cells increases during OA pathogenesis. Some of these clusters consist of both CD166+ and CD166− cells. This suggests that OA-MSCs, which are CD166+, may also give rise to chondrocytes, which are CD166−. Since OA-MSCs contain the hallmarks of OA, reproduction of OA-MSCs, as occurred in cell clusters, could sustain degenerative phenotypes in OA cartilage.

Our study suggests that it would be beneficial to consider inhibiting the activation of such OA cartilage-derived stem cells as a novel therapeutic approach for preventing disease progression. Indeed, OA-MSC lines generated in this study may be instrumental for testing the *in vitro* efficacy of potential anti-OA drugs for this purpose. We found that knocking down RUNX2 alone is insufficient for suppressing type X collagen expression in either type of OA cartilage-derived stem cell. Furthermore, knocking down RUNX2 activated MMP-13 expression. This is markedly different from chondrocytes in which knocking down RUNX2 sufficiently inhibits hypertrophic genes including type X collagen and MMP-13. Our findings suggest that the gene regulatory network is altered in OA-MSCs. Since NOTCH1 has been shown to play a role in OA pathogenesis^45–47^, we also tested the effect of NOTCH1 knockdown on OA-MSCs. While NOTCH1 knockdown alone does not have any effect, knocking down both RUNX2 and NOTCH1 significantly suppressed COL10A1 and ADAMTS5 mRNA levels in both types of OA-MSCs. However, the MMP-13 mRNA levels remained elevated. Therefore, the gene regulatory pathways need to be further analyzed in OA-MSCs. Such studies may be important for developing therapies for OA by targeting OA-MSCs.

Stable OA-MSC cell lines are useful, consistent, and convenient resources for analysis and drug testing for OA treatment in different laboratories, since primary human OA cartilage stem cells can be heterogeneous, early senescent, or unable to divide beyond a certain number of population doublings^21^. However, immortalizing OA-MSCs may introduce a notable limitation by altering cellular properties. Although we confirmed that primary OA-MSCs and immortalized lines exhibit similar expression of selective markers, it is possible that T-Antigen mediated immortalization may introduce deviations in other untested markers. Furthermore, the lack of available normal healthy human cartilage to compare with our findings in OA cartilage remains a limitation of this study.

In summary, we characterized the properties of osteoarthritis cartilage derived mesenchymal stem cells (OA-MSCs) in this study. Based on our analysis, we propose the following working hypothesis. OA-MSCs are cells found within articular cartilage or other joint tissues in OA patients, which possess some characteristics associated with mesenchymal stem cells, specifically the ability to give rise to heterogeneous cell types found in OA cartilage. OA-MSCs may mediate OA pathogenesis through the process of stem cell differentiation into multiple cell types. OA-MSCs may persist in OA tissues as a distinct population and cause failure of OA treatment by giving rise to OA tissue hallmarks such as chondrocyte hypertrophy, matrix proteases release, cell senescence, and osteophyte formation upon activation by mechanical, chemical or biological stress. We suggest that understanding of the novel properties of OA-MSCs and development of specific therapies targeted at OA-MSCs may be critical for improving treatment and life quality of OA patients. The stable human OA-MSC cell lines described here can be a powerful resource for analysis, understanding, and developing treatment of human OA.

## Experimental Procedures

### Patients

Tissues were acquired with informed patient consent. All experiments and protocols were approved by and used in accordance with the Institutional Review Board (IRB) of Rhode Island Hospital (RIH). RIH is an institution that is in compliance with the International Conference on Harmonization and Good Clinical Practice (ICH GCP) as they correspond to the FDA/DHHS regulations. OA-MSCs were isolated from human osteoarthritic articular cartilage. Cells from a male (61) and female patient (69) undergoing total knee replacement surgery were pooled together in order to generate multiple cell lines. Human chondrocytes utilized in this study were freshly isolated from OA articular cartilage obtained from complete joint replacement surgeries. OA cartilage-derived cells were isolated from the full thickness of articular cartilage and did not contain lesions or exhibit tissue discoloration.

### Cell culture

Dulbecco’s Modified Eagle Medium (DMEM), fetal bovine serum (FBS), Hank’s Balanced Salt Solution (HBSS) and Penicillin Streptomycin (Pen Strep) were purchased from Life Technologies, Grand Island, NY. Chondrocytes were grown using DMEM supplemented with 10% FBS and 1% Pen Strep. Primary OA-MSCs and OA-MSC lines were maintained in DMEM supplemented with 10% FBS, 1% Pen Strep, 100 mM HEPES, 2 mM L-glutamine, 0.1 mM ascorbic acid, 0.1 mM sodium pyruvate, 2.7 µM L-glucose *(DMEM*+*)*. All cells were grown in a 37 °C cell culture incubator.

### Isolation and enrichment of OA cartilage-derived stem cells

Cells were isolated from the entire thickness of articular cartilage derived from osteoarthritic knees. Samples were washed three times with 1xHBSS diced into small fragments. The diced cartilage tissue was treated with Pronase (Roche, Indianapolis, IN, USA) in 1xHBSS (2.0 mg/mL) for 30 minutes in a 37 °C shaking water bath. Cartilage fragments were then washed twice with DMEM and further digested with Type IA Crude Bacterial Collagenase (Sigma-Aldrich, St. Louis, MO, USA) (1.0 mg/mL) for 8 hrs in a 37 °C shaking water bath. Cells were strained through a 100 µm nylon cell strainer (BD, Franklin Lakes, NJ, USA) to remove clumps and washed three times with 5.0 mL of DMEM supplemented with 10% FBS. Cells were then counted using a hemacytometer. Cartilage-derived stem cells were enriched using differential cell adhesion to fibronetin similar to a previously described method^11^. Cells (2000 cells/mL) were plated in 60 mm dishes that had been coated at 4 °C overnight with 10 µg/mL of fibronectin in 0.1 M PBS containing 1.0 mM MgCl and 1.0 mM CaCl_2_. Cells were seeded and left for 20 min at 37 °C. After 20 min, non-adherent cells were removed from the plates and fresh *DMEM*+ was added. Adherent cells were observed and counted under a light microscope. After 10 days, single cells that had formed individual colonies consisting of ≥32 cells were isolated using glass cloning cylinders (Sigma-Aldrich, St. Louis, MO, USA), taking care not to cross contaminate with cells from neighboring regions, and reseeded in individual wells of 6-well cell culture plates. Colonies were cultured for one week.

### Generation of stable OA cartilage-derived stem cell lines

After one week in culture, these clonally derived OA-MSC colonies were treated with a retroviral vector pRetro-E2 SV40 (Applied Biological Materials Inc., Richmond, BC, Canada). According to the manufacturer’s instructions, colonies were then continuously expanded for two months (>20 passages) until only the successfully transformed cells remained. Each stem cell line was genotyped/profiled for authenticity using autosomal short tandem repeat (STR) loci analysis (Genetica DNA Laboratories, Burlington, NC, USA).

### Real-time PCR

Gene expression analysis was conducted using real-time PCR. Total messenger RNA (mRNA) was isolated from cells using a RNAqueous Kit (Ambion, Austin, TX, USA) according to manufacturer’s instructions. mRNA was reverse transcribed using iScript cDNA Synthesis Kit (Bio-Rad, Hercules, CA, USA) according to the manufacturer’s instructions. Supplementary Table 2 lists forward and reverse primer sequences used to conduct gene expression analysis. Ribosomal RNA (rRNA) 18 S was used as the housekeeping gene for normalization. mRNA transcript levels were calculated using the delta delta Ct (∆∆Ct) method, normalized to rRNA 18 S expression as follows: X = 2 − ∆∆Ct, in which ∆∆Ct = (CtExp − Ct18S) − (CtCtl − Ct18S) and X = Relative transcript; CtCtl = Ct of control group.

### Flow cytometry

Pre-conjugated antibodies CD49c-APC and CD166-PE were purchased from BioLegend, San Diego, CA, USA. SSEA4-PE, CD29-APC, CD54-PE, CD90-FITC, CD105-APC, CD106-APC were purchased from Miltenyi Biotec Inc., San Diego, CA, USA. Isotype IgG control antibodies were also purchased from Miltenyi Biotec Inc. Cells to be stained were washed 2 times with 5.0 mL of sterile HBSS and detached using 2.0 mL of TrypLE Express (Life Technologies, Grand Island, NY, USA). Cells were washed with DMEM supplemented with 10% FBS and spun down using a centrifuge set for 300xg. Cells were washed once again with 5.0 mL sterile 1x PBS and spun down at 300xg. Viable cell number was quantified using a hemacytometer and 0.4% Trypan blue solution (Life Technologies, Grand Island, NY, USA). For each sample to be stained, 1.0 × 10^6^ viable cells were resuspended in 100 µL of Flow buffer (1x PBS, pH 7.2, 0.5% bovine serum albumin and 2 mM EDTA). Pre-conjugated antibody (10 µL) was added to the resuspension, mixed and incubated for 10 min in the dark at 4 °C. Cells were washed 3 times with 1.0 mL of 1x PBS and resuspended in 500 mL of Flow buffer before single channel FACS analysis using an Accuri C6 Flow Cytometer (BD Biosciences, San Jose, CA, USA). Control experiments for non-specific staining using mouse IgG were performed alongside all experiments.

### Differentiation assays

OA-SC lines were assessed for their chondrogenic, osteogenic and adipogenic differentiation potential. For chondrogenesis, 2.5 × 10^5^ viable cells were centrifuged at 300xg for 10 min in a 15 mL conical tube. The cell pellet was cultured in 1.0 mL of Stempro ® Chondrocyte Differentiation Media (Life technologies, Grand Island, NY, USA) containing gentamicin (5.0 µg/mL). Media was changed every 3 days making sure not to disturb cell pellet. After 21 days, cell pellets were fixed in formalin, paraffin embedded and sectioned into 3.0 µm thick sections. The sections were mounted onto slides, cleared with xylene and rehydrated using sequential incubation in 100%, 95%, 70% and 50% ethanol solution prior to staining with Safranin-O. Images of pellet sections were taken using a Nikon Eclipse 90i microscope at 20x magnification. For osteogenesis, 5.0 × 10^3^ viable cells were seeded into single wells of 12-well cell culture plates and cultured using Stempro ® Osteogenesis differentiation media (Life technologies, Grand Island, NY, USA) containing gentamicin (5.0 µg/mL) according to the manufacturer’s instructions. For osteogenesis, 5.0 × 10^3^ viable cells were seeded into single wells of 12-well cell culture plates. Media was changed every 3–4 days and cells were stained using Alizarin Red after 21 days in monolayer culture. Images were taken using a Leica MZ6 dissecting microscope at 4x magnification. For adipogenesis, 5.0 × 10^4^ cells were seeded into a single well of a 6-well plate and cultured using Stempro ® Adipogenesis differentiation media (Life technologies, Grand Island, NY, USA) containing gentamicin (5.0 µg/mL) according to the manufacturer’s instructions. Media was changed every 4 days and cells were stained Oil Red-O and hematoxylin after 21 days in monolayer culture. Images were taken using a Nikon Eclipse TE2000 inverted microscope at 20x magnification.

### Gene knockdown

NOTCH1 and/or RUNX2 genes were transiently knocked down using Lipofectamine 2000 transfaction (Thermo Fisher, Grand Island, NY, USA) reagent and human gene specific ON-TARGET plus siRNA (GE Dharmacon, Lafayette, CO, USA). Cells were plated in 12-well cell culture plates at a density of 1.0 × 10^5^ per well and transfected according to manufacturer’s instructions.

### Immunohistochemistry

Human OA cartilage sections were fixed overnight in formalin solution and paraffin embedded. The blocks were then sectioned (3.0 µm thick), mounted onto slides, cleared with xylene and rehydrated using sequential incubation in 100%, 95%, 70% and 50% ethanol solution. Sample slides were rinsed in deionized water and antigen retrieval was performed using sodium citrate buffer (10 mM sodium citrate, pH 6) and an 850 W microwave. Slides were blocked overnight at 4 °C using 1% bovine serum albumin in 1x PBS to eliminate non-specific binding of the primary antibody. Slides were stained with a monoclonal mouse antibody (diluted 1:100 in 1x PBS, 1% BSA) against human CD166 (Abcam, Cambridge, MA, USA), overnight at 4 °C. Sections were then stained for 30 min with a green fluorescently labeled anti-mouse secondary antibody Alexa Fluor ab150105 (Abcam, Cambridge, MA, USA). Fluorescent images were acquired at 20x magnification using a Nikon Eclipse 90*i* Digital Imaging System.

### Protein analysis

Cells were washed with PBS and then lysated using M-PER lysis buffer (Pierce, Illinois, USA) containing protease inhibitors for 30 min on ice with constant agitation. The lysates were centrifuged at 12,000 × g for 15 minutes at 4 °C. Supernatants were used to determine protein concentrations using BCA assay (Pierce, Illinois, USA). Samples were heated at 95 °C, for 5 minutes, and separated by 10% SDS-polyacrylamide gel. Blots were transferred to polyvinylidene difluoride membrane (Whatman, USA) for 70 minutes at a constant voltage of 100 V. The membrane was blocked for 1 hour with 5% bovine serum albumin in Tris-buffered saline-Tween 20 (0.1%) (TBS-T), at 25 °C. The membrane was incubated with antibodies against SOX9, type X collagen or Tubulin (loading control) overnight, at 4 °C. All primary antibodies were purchased from Abcam, USA. The membrane was rinsed 5 times (10 minutes each time) with TBS-T. The membrane was incubated with anti-rabbit-Alexa Fluor 680 (Molecular Probes, Eugene, OR, USA) for 1 hour, at 25 °C. Membranes were imaged using an Odyssey fluorescence scanner (LI-COR Biosciences, Lincoln, NE, USA). The detection of the MMP-13 protein in medium of OA-MSC2 and OA-MSC18 treated with basal medium or chondrogenic differentiated medium ate day 3, 7 and 14 was performed using a MMP13 Human ELISA Kit (Thermo Fisher, USA) according to manufacturer instructions. Optical density (OD) values were quantified using a SpectraMAX Me2 microplate reader (Molecular Devices, Sunnyvale, CA).

### Statistics

Statistics were performed using a Student’s t-test when analyzing two groups or one-way analysis of variance (ANOVA) followed by post-hoc analysis when analyzing more than two groups. Error bars represent ± one standard deviation of the mean. P-values smaller or equal to 0.05 were considered statistically significant.

## Electronic supplementary material

Supplementary Materials
