# Supplementary material for: Molecular characterization of mesenchymal stem cells in human osteoarthritis cartilage reveals contribution to the OA phenotype
Source: Sci Rep. 2018 May 4;8:7044. doi: 10.1038/s41598-018-25395-8 (PMC5935742; doi:10.1038/s41598-018-25395-8)
Supplement: Supplementary file 1 — Supplementary Materials [file 41598_2018_25395_MOESM1_ESM.pdf]

SUPPLEMENTARY MATERIALS

Title:

Molecular characterization of mesenchymal stem cells in human osteoarthritis cartilage reveals contribution to the OA phenotype

Authors:

Chathuraka T. Jayasuriya (PHD)[2], Nan Hu (MD)[1,2] Jing Li (PHD)[1,2], Nicholas Lemme (BS)[2], Richard Terek (MD)[2] Michael G. Ehrlich (MD) [2] and Qian Chen (PHD)[1,2,*].

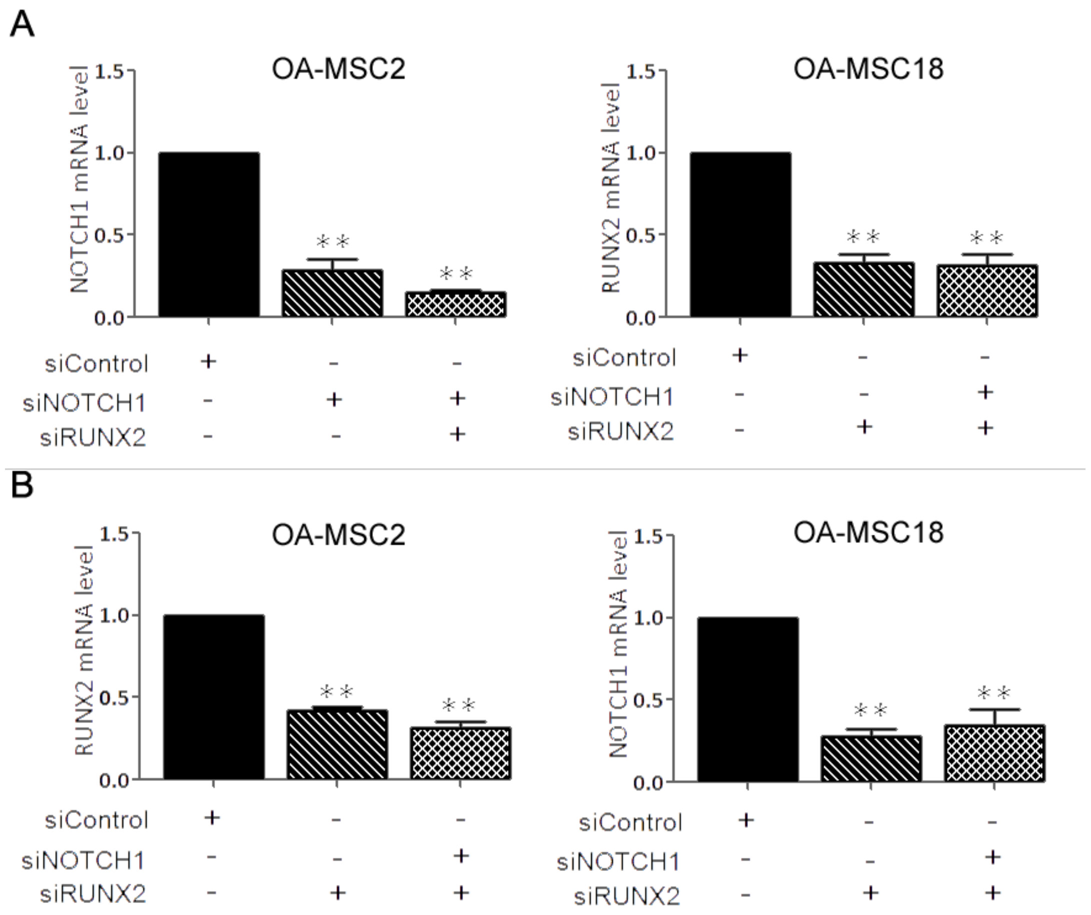

Supplementary Figure 1. The knockdown efficiency of RUNX2 and NOTCH1 in OA-MSC2 and OA-MSC18. n≥3. ** p≤0.01, relative to scrambled siRNA control transfected group. Data are represented as mean ±SD.

Supplementary Table. 1

Line	D3S1358	D21S11	D18S51	Penta E	D5S818	D13S317	D7S820	D16S539	CSF1PO
OA-MSC1	16	29	13, 21, 22	12	9, 11	9, 11	8, 10	11	10, 11
OA-MSC2	16	29	13, 21	12	9, 11	9, 11	8, 10	11	10, 11
OA-MSC5	16	29	13, 21	12	9, 11	9, 11	8, 10	11	10, 11
OA-MSC6	16	29	13, 21	12	9, 11	9, 11	8, 10	11	10, 11
OA-MSC8	16, 18	28, 32.2	19	10, 14	11	9, 12	8, 11, 11.1	10, 12	12, 13
OA-MSC12	16	29	13, 21	12	9, 11	9, 11	8, 10	11	10, 11
OA-MSC13	16, 18	28, 32.2	13, 21	10, 14	11	9, 12	8, 11, 11.1	10, 12	12, 13
OA-MSC14	16, 18	28, 32.2	19	10, 14	11	9, 12	8, 11, 11.1	10, 12	12, 13
OA-MSC18	16	29	13, 21	12	9, 11	9, 11	8, 10	11	10, 11

Line	Penta D	vWA	D8S1179	TPOX	FGA	AMEL
OA-MSC1	9, 11	14, 19	12, 14	8, 12	20, 21	X
OA-MSC2	9, 11	14, 19	12, 14	8, 12	20, 21	X
OA-MSC5	9, 11	14, 19	12, 14	8, 12	20, 21	X
OA-MSC6	9, 11	14, 19	12, 14	8, 12	20, 21	X
OA-MSC8	11, 12	17, 19	8, 14	8, 10	19, 21	X, Y
OA-MSC12	9, 11	14, 19	12, 14	8, 12	20, 21	X
OA-MSC13	11, 12	17, 19	8, 14	8, 10	19, 21	X, Y
OA-MSC14	11, 12	17, 19	8, 14	8, 10	19, 21	X, Y
OA-MSC18	9, 11	14, 19	12, 14	8, 12	20, 21	X

Supplementary Table 1. Patient origin of each OA-MSC line. Each cell line was profiled in order to determine their patient origin using autosomal short tandem repeat (STR) loci and the amelogenin locus. Profile results confirmed that cell lines 1, 2, 5, 6, 12 and 18 originated from an individual female patient. Cell lines 8, 13 and 14 originated from an individual male patient. OA-MSC1 appears to have an allele difference in D18S51 locus compared to the rest of the lines from the same patient. This difference is likely due to genetic instability common in transformed cells.

Supplementary Table. 2

Human gene	RefSeq Accession	Forward Seq.	Reverse Seq.
ACAN	NM_001135.3	5'-ACCAGACGGGCCTCCCAGAC-3'	5'- ACAGCAGCCACACCAGGAAC-3'
COL2A1	NM_001844.4	5’-TGAGGGCGCGGTAGAGACCC-3’	5’-TGCACACAGCTGCCAGCCTC-3’
CD49e	NM_002205.4	5’-GGCTTCAACTTAGACGCGGA-3’	5’- ATTCAATGGGGGTGCACTGT-3’
SOX9	NM_000346.3	5’-GGACCAGTACCCGCACTTGCA-3’	5’- GTTCTTCACCGACTTCCTCCGCCG-3’
MATN3	NM_002381.4	5’-TGAGGGCTACACTCTGAATGC-3’	5’-GTGCTTCCTCAGTGGCTGAA-3’
PRG4	NM_001127708.2	5’-TTCATCTCAAGAGCTTTCCTGT-3’	5’-TGATGGTTTGAGATGCTCCTG-3’
PRX1	NM_006902.4	5’-CATCGTACCTCGTCCTGCTC-3’	5’-GTAAACAACATCTTGGGAGGGA-3’
LPL	NM_000237.2	5’-GACACTTGCCACCTCATTCC-3’	5’-AGCCATGGATCACCATGAAGG-3’
COL1A1	NM_000088.3	5’-CAGGAGGCACGCGGAGTGTG-3’	5’-GGCAGGGCTCGGGTTTCCAC-3’
ALPL	NM_000478.5	5’-CTGGACGGACCCTCGCCAGTG-3’	5’- TGCAATCGACGTGGGTGGGAGG-3’
RUNX2	NM_001015051.3	5’-GGCTGCAAGCAGTATTTACAACA-3’	5’-CGGATCCCAAAAGAAGTTTTGCT-3’
IHH	NM_002181.3	5’-GGAGAACACAGGCGCCGACC-3’	5’-CGGTCACCCGCAGCTTCACA-’3
HHIP	NM_022475.2	5’-CCCCCAACCCAACTGACACTG-3’	5’-CGGGGCTGTAGGGCACTCC-3’
COL10A1	NM_000493.3	5’-GCCCACAGGCATAAAAGGCCC-3’	5’-GAAGGACCTGGGTGCCCTCGA-3’
NOTCH1	NM_017617.4	5’-CTCCTACCGCTGCGTCTG-3’	5’-GGCAGGCAGGCACACT-3’
MMP-13	NM_002427.3	5’-ATGCGGGGTTCCTGATGTGG-3’	5’-GGCCCAGGAGGAAAAGCATG-’3
ADAMTS5	NM_007038.4	5’-GGCCGTGGTGAAGGTGGTGG-3’	5’-GCTGCGTGGAGGCCATCGTC-3’

Supplementary Table 2. Forward and reverse sequences of primers used to conduct Real-time RT-PCR for gene mRNA expression analysis.
